# Point-of-care ultrasound to assess volume status and pulmonary oedema in malaria patients

**DOI:** 10.1007/s15010-021-01637-2

**Published:** 2021-06-10

**Authors:** Christina M. Pugliese, Bayode R. Adegbite, Jean R. Edoa, Ghyslain Mombo-Ngoma, Fridia A. Obone-Atome, Charlotte C. Heuvelings, Sabine Bélard, Laura C. Kalkman, Stije J. Leopold, Thomas Hänscheid, Ayola A. Adegnika, Mischa A. Huson, Martin P. Grobusch

**Affiliations:** 1 Center of Tropical Medicine and Travel Medicine, Department of Infectious Diseases, Amsterdam University Medical Centers, Location AMC, Amsterdam Infection and Immunity, Amsterdam Public Health, University of Amsterdam, Amsterdam, The Netherlands; 2 School of Medicine and Health Sciences, The George Washington University, Washington, DC USA; 3 Centre de Recherches Médicales de Lambaréné (CERMEL), Lambaréné, Gabon; 4 Institute for Tropical Medicine, German Center for Infection Research (DZIF), University of Tübingen, Tübingen, Germany; 5 Department of Tropical Medicine, Bernhard Nocht Institute for Tropical Medicine, Department of Medicine, University Medical Centre, Hamburg-Eppendorf, Hamburg, Germany; 6 Department of Paediatric Pulmonology, Immunology and Intensive Care Medicine, Charité-Universitätsmedizin Berlin, Berlin, Germany; 7 Berlin Institute of Health, Berlin, Germany; 8 Faculdade de Medicina, Universidade de Lisboa, Lisboa, Portugal; 9 Department of Medical Microbiology and Infectious Diseases, Erasmus Medical Center, Rotterdam, The Netherlands; 10 Masanga Medical Research Unit, Masanga, Sierra Leone; 11 Institute of Infectious Diseases and Molecular Medicine, University of Cape Town, Cape Town, South Africa

**Keywords:** Fluid management, Gabon, Severe malaria, Point-of-care ultrasound, Volume status

## Abstract

**Purpose:**

Fluid management is challenging in malaria patients given the risks associated with intravascular fluid depletion and iatrogenic fluid overload leading to pulmonary oedema. Given the limitations of the physical examination in guiding fluid therapy, we evaluated point-of-care ultrasound (POCUS) of the inferior vena cava (IVC) and lungs as a novel tool to assess volume status and detect early oedema in malaria patients.

**Methods:**

To assess the correlation between IVC and lung ultrasound (LUS) indices and clinical signs of hypovolaemia and pulmonary oedema, respectively, concurrent clinical and sonographic examinations were performed in an observational study of 48 malaria patients and 62 healthy participants across age groups in Gabon.

**Results:**

IVC collapsibility index (CI) ≥ 50% on enrolment reflecting intravascular fluid depletion was associated with an increased number of clinical signs of hypovolaemia in severe and uncomplicated malaria. With exception of dry mucous membranes, IVC-CI correlated with most clinical signs of hypovolaemia, most notably sunken eyes (*r* = 0.35, *p* = 0.0001) and prolonged capillary refill (*r* = 0.35, *p* = 0.001). IVC-to-aorta ratio ≤ 0.8 was not associated with any clinical signs of hypovolaemia on enrolment. Among malaria patients, a B-pattern on enrolment reflecting interstitial fluid was associated with dyspnoea (*p* = 0.0003), crepitations and SpO_2_ ≤ 94% (both *p* < 0.0001), but not tachypnoea (*p* = 0.069). Severe malaria patients had increased IVC-CI (*p* < 0.0001) and more B-patterns (*p* = 0.004) on enrolment relative to uncomplicated malaria and controls.

**Conclusion:**

In malaria patients, POCUS of the IVC and lungs may improve the assessment of volume status and detect early oedema, which could help to manage fluids in these patients.

**Supplementary Information:**

The online version contains supplementary material available at 10.1007/s15010-021-01637-2.

## Background

Malaria remains one of the top infectious disease killers globally. Of the 229 million cases reported in 2020, 409,000 resulted in death. Sub-Saharan Africa carries most of the global malaria burden, accounting for about 94% of cases and deaths [1].

Central to the pathogenesis of severe falciparum malaria is the sequestration of parasitised red blood cells, leading to microcirculatory obstruction, endothelial dysfunction and impaired tissue perfusion [2, 3]. Accordingly, metabolic (i.e. lactic) acidosis and renal impairment are among the strongest predictors of death in malaria patients across age groups [4, 5].

Fluid management in malaria patients is challenging due to the fragile balance between intravascular fluid depletion and induction of pulmonary oedema. Dehydration, on the one hand, is common owing to fluid losses from fever, vomiting, sweating and impaired consciousness over several days. Consequently, studies show that both adults and children with severe malaria are intravascularly fluid-depleted [6, 7], although liberal fluid resuscitation has not been shown to improve acid–base status or renal function in adult patients [6]. Although this suggests that the primary driver of acidosis is microvascular obstruction rather than hypovolaemia [2], a recent study showed that hypovolaemia reduced cardiac index reserve in adults with severe malaria, which may impact survival by increasing susceptibility to shock [8]. Markers of impaired perfusion are also well established in paediatric malaria and are associated with increased mortality [9, 10].

On the other hand, liberal fluid resuscitation may induce or aggravate pulmonary oedema and acute respiratory distress syndrome (ARDS). In the setting of severe malaria, pulmonary oedema might be caused by increased vascular permeability in the lungs due to capillary leakage and endothelial damage following sequestration of late-stage *Plasmodium falciparum* infected red blood cells. The pathophysiology of malaria itself; thereby, increases the risk for iatrogenic fluid overload and subsequent pulmonary oedema [11]. In patients with severe falciparum malaria in Bangladesh, for instance, studies show that young, previously healthy adults who would normally be expected to cope with a fluid bolus can still develop pulmonary oedema, albeit a rare complication [6]. In adults, respiratory complications often develop late in the disease course, progress rapidly and are often fatal [11]. Furthermore, rapid administration of bolus fluids increased mortality at 48 h in the Fluid Expansion as Supportive Therapy (FEAST) trial, which included a large cohort of febrile African children (57% falciparum malaria) with compensated shock [12]. A recent re-analysis of the data suggests that worsening respiratory function from pulmonary oedema was a main contributor to excess deaths in bolus recipients [13].

Improved fluid therapy guidelines are needed for clinicians treating patients with severe malaria. This is particularly true in settings with limited access to mechanical ventilation for patients who develop severe respiratory complications [14]. Given that most patients with malaria are treated in resource-poor areas, the World Health Organization (WHO) recommends frequent clinical evaluation of volume status to guide fluid therapy (e.g. jugular venous pressure, urine output, peripheral perfusion, capillary refill and skin turgor) [15]; however, clinical assessment of volume status is challenging, as no individual clinical sign has demonstrated adequate sensitivity or specificity [16–18].

Ultrasound (US), including inferior vena cava (IVC) and lung ultrasound (LUS), is an alternative means to assess volume status. The size and dynamics of the IVC, a highly compliant vessel, vary with changes in right atrial (RAP) and central venous pressures (CVP) and intravascular volume [19]. The degree of IVC collapsibility measured with US has been shown to correlate well with intravascular volume in adult and paediatric patients [19–21]. In addition, several studies have identified the IVC-to-aorta ratio (IVC/Ao) as a promising means of evaluating intravascular volume status in adults and children [22–24].

**Fig. 1:**
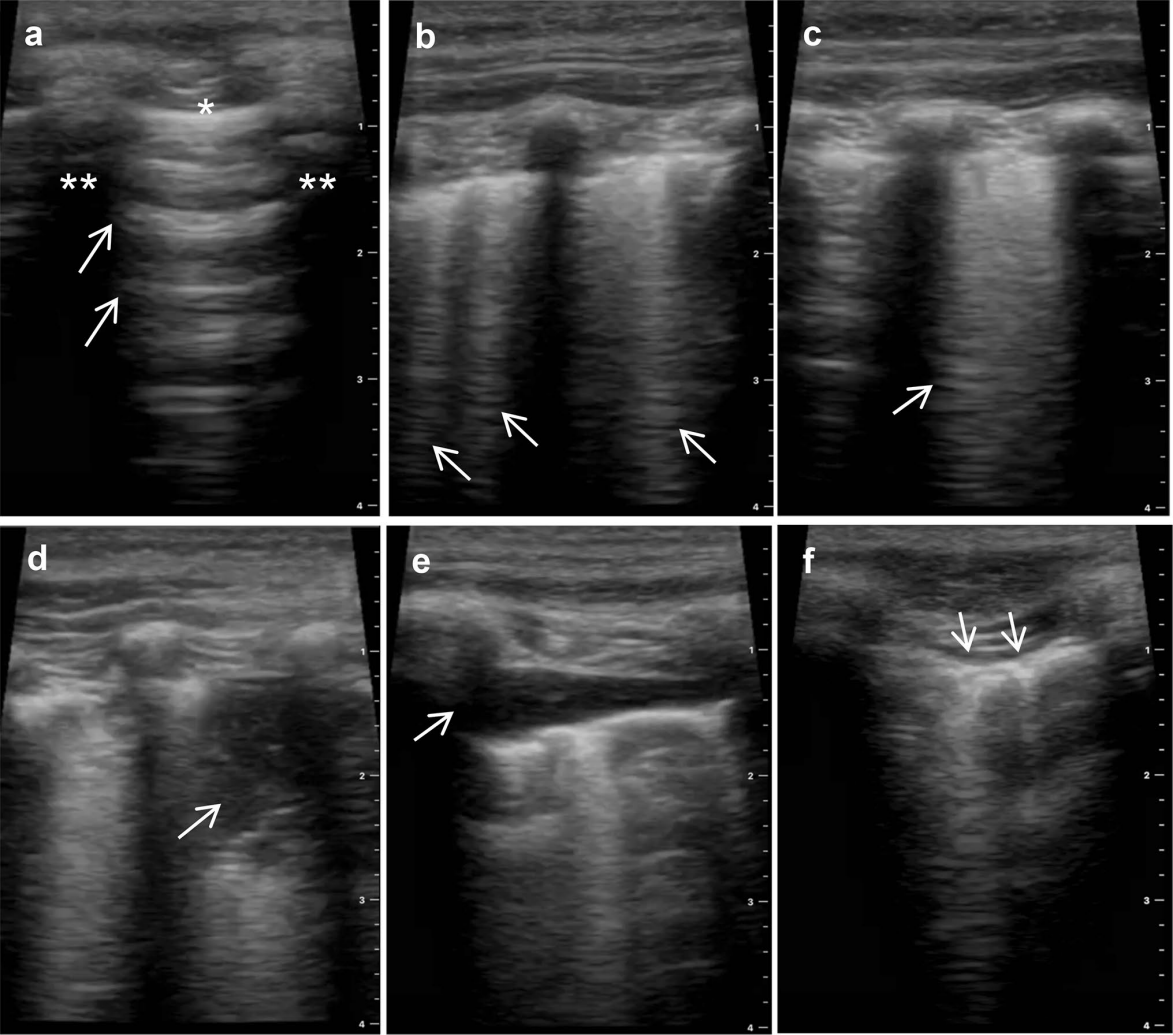
Lung ultrasound patterns. **a** A lines (*arrows*), reverberation artefacts of the pleural line (*) between two rib shadows (**); **b** Isolated B lines (*arrows*), pathological signs of interstitial pulmonary oedema; **c** Multiple coalescing B lines (*arrow*); **d** Consolidation (*arrow*) showing characteristic tissue-like pattern (i.e. “lung hepatisation”) and air bronchograms; **e** Pleural effusion (*arrow*); **f** Sub-pleural nodules (*arrows*)

Point-of-care LUS is a non-invasive, sensitive tool for evaluating lung aeration (Fig. 1). Normal lung aeration is indicated by the presence of A-lines with normal lung sliding, while multiple B-lines (i.e. hyper-echoic, ‘laser-like’ artefacts that arise at the pleural line and continue to the edge of the screen without fading) are a sign of interstitial fluid [25, 26]. Inaddition, LUS can be used to detect lung consolidations, which present with characteristic tissue-like patterns (i.e. ‘lung hepatisation’) and dynamic air bronchograms [26]. Combining LUS patterns into composite outcome measures have proven useful for detection of interstitial fluid and pulmonary oedema [27, 28].

In light of the recent technological advances, portable and handheld US devices are now widely available and relatively affordable. Point-of-care ultrasound (POCUS) has thus been increasingly adopted as a non-invasive tool to guide diagnosis, management and treatment in a variety of settings, including several tropical infectious diseases [29]. In the setting of malaria, IVC US could help identify patients with hypovolaemia who may benefit from fluid therapy. In addition, a recent study found abnormal LUS patterns in 42% of adult malaria patients on admission, suggesting that LUS may help with early detection of pulmonary oedema [28]. Earlier studies also show that increased extravascular lung water as measured by thermodilution is often present before physical signs of pulmonary oedema are detectable, highlighting the need for a more sensitive and non-invasive measure to assess pulmonary oedema [16, 30]. The high sensitivity of US may therefore prove to be a crucial tool to guide fluid management and to prevent fluid overload in these patients.

We hypothesised that POCUS of the IVC and lungs correlates with clinical signs of hypovolaemia and pulmonary oedema, respectively. We also hypothesised that pulmonary oedema would be seen more frequently in patients with limited IVC collapsibility, indicating fluid overload.

## Methods

### Study design and setting

We conducted a prospective proof-of-concept observational study of patients of all ages with malaria, with age-matched healthy volunteers as a control group. The study was conducted from September to December 2019 in Lambaréné, a town of approximately 39,000 inhabitants in Moyen-Ogooué Province of Gabon, in the Central African rainforest region. Patients were recruited from the paediatrics and internal medicine wards of the Albert Schweitzer Hospital (HAS), a 150-bed general hospital that serves the local population of Lambaréné and the surrounding villages. The hospital has both inpatient and outpatient services in emergency medicine, internal medicine, paediatrics, surgery and obstetrics/gynaecology. It operates as a private non-governmental organization (NGO) with joint funding from the Gabonese Ministry of Health.

### Ethical considerations

The study was approved by the Scientific Review Committee of the Centre de Recherches Médicales de Lambaréné (CERMEL). Ethical approval for the study was obtained from the Ethics Review Committee of CERMEL (Reference number: CEI-CERMEL 10/2019). The study was also endorsed by the Ethics Committee of the Amsterdam University Medical Centers of the University of Amsterdam, the Netherlands. Written informed consent was obtained from all participants or their legal representative (if less than 18 years of age) prior to enrolment. The study was conducted in accordance with Good Clinical Practice guidelines [31].

### Inclusion and exclusion criteria

Consecutive consenting patients (in paediatric patients—their legal representative) of all ages with a diagnosis of malaria were included. Malaria was confirmed through detection of asexual parasitaemia of any *Plasmodium* species on light microscopy, with parasitaemia quantified according to the Lambaréné method [32], in a patient with fever (measured tympanic temperature ≥ 38.0 °C or reported history of fever during the past 24 h).

Severity of the disease was categorised according to the following modified WHO criteria [15]: (1) impaired consciousness, defined as prostration (i.e. inability to sit or stand unassisted or inability to breastfeed in children less than eight months of age) or coma (i.e. inability to localise a painful stimulus); (2) repeated convulsions; (3) severe anaemia (haemoglobin < 7 g/dL in adults/ ≤ 5 g/dL in children under 12 years; (4) hypoglycaemia (blood glucose < 2.2 mmol/L); (5) peripheral asexual stage parasitaemia > 500,000/µL; (6) shock (systolic blood pressure [SBP] < 80 mmHg in adults/ < 70 mmHg in children under 12 years, with impaired perfusion (i.e. capillary refill time [CRT] ≥ 3 s or cool extremities); (7) pulmonary oedema (oxygen saturation < 92% on room air with respiratory rate > 30/min).

Age-matched healthy volunteers were recruited from the local community.

### Study procedures

On enrolment, baseline demographic and medical history data were collected from the patient or caregiver. Routine hospital venous blood samples were collected in all patients for full blood count analyses and malaria microscopy. Clinical and sonographic data were measured concurrently at the point-of-care prior to initiating anti-malarial treatment and repeated on hospital days 1, 2 and 3. Patients with uncomplicated malaria were treated with either artesunate–amodiaquine or artemether–lumefantrine. Patients with severe malaria were treated by the attending physician with intravenous artesunate and standard supportive care in accordance with WHO guidelines [15]. Intravenous fluids were provided at the discretion of the attending physician according to the local standard of care. To assess the relationship between fluid administration and US parameters, intravenous fluid intake was recorded daily.

Among the control group, clinical and sonographic assessments were performed only at baseline, either at home or at the hospital’s research centre. Screening for malaria was not performed in this group.

### Clinical assessment

The oral mucous membranes were classified as moist or dry. Eyes were classified as normal or sunken. Capillary refill time (CRT) was assessed by applying ten seconds of pressure to the nailbed of the distal phalanx and was classified as normal (< 2 s), prolonged (2–3 s) or very prolonged (≥ 3 s). Tissue turgor was examined by pinching the skin of the abdomen and was classified as normal (flattens immediately) or decreased (flattens slowly). The extremities were palpated from the dorsum of the foot to the knee and classified as cold (noticeable temperature gradient from cold to warm) or normal. Peripheral perfusion was assessed by palpating the dorsalis pedis pulses bilaterally and classified as weak or normal. Respiratory effort was classified as increased (noticeably laboured breathing) or normal. The lungs were auscultated and crepitations were classified as present or absent. Oxygen saturation (SpO_2_) and pulse rate were recorded using a pulse oximeter (Zacurate^®^ 500BL, Stafford, TX, USA). Tachycardia was defined as a heart rate (HR) > 160 beats per minute (bpm) in children younger than 12 months, > 140 bpm in children 1–2 years, > 120 in children 3–5 years, > 118 in children 6–11 years and > 100 in participants aged 12 years or older. Severe tachycardia was defined as a heart rate > 180 bpm in children younger than 12 months, > 160 bpm in children 1–5 years, > 140 bpm in children 6–11 years and > 120 bpm in participants aged 12 years or older. Blood pressure was measured using a manual sphygmomanometer (Diagnostix^®^ 700, Hauppauge, NY, USA). Hypotension was defined as a systolic blood pressure (SBP) < 70 mmHg in children one month to less than 12 months of age, < 70 + (age in years × 2) mmHg in children one to less than ten years of age and < 90 mmHg in participants ten years of age or older. Respiratory rate was counted at the bedside for 30 s. Tachypnoea was defined as a respiratory rate (RR) > 60/min in children less than 12 months, > 40/min in children 1–3 years of age, > 34/min in children 3–6 years of age or > 30/min in participants six years of age or older [33].

### US scanning protocol

US scans were performed by a senior medical student (CMP) who had attended a 2-week POCUS training course; onsite supervision was available when needed. For this study, a portable US machine was used (Butterfly IQ™, Guilford, CT, USA), which is equipped with a 2D array microsensor technology capable of emulating all transducer types (i.e. linear, curved or phased array). The device includes separate pre-programmed settings for adults or children, optimized for abdominal or chest applications. For practical purposes, most patients were scanned in the supine position, whilst small children were scanned in a seated position in the parent’s lap for improved compliance. Following each examination, the sonographer assessed the overall compliance of participants as ‘good’ (calm and cooperative), ‘moderate’ (agitated but remained cooperative) or ‘poor’ (agitated and uncooperative/moving throughout examination). All US images were stored for post hoc analysis.

#### IVC US protocol

US measurements of the IVC and aorta diameters were obtained in the sub-xiphoid region. The IVC was assessed in the longitudinal plane approximately 2 cm distal to the IVC–hepatic vein junction. M-mode was used to capture a cine loop over a normal respiratory cycle. The maximal and minimal anterior–posterior (A–P) IVC diameters were measured from inner wall to inner wall. The maximal A–P aorta diameter was measured in the transverse plane from inner wall to inner wall.

#### LUS protocol

In participants 12 years of age or older, the scanning protocol described by Leopold et al. was employed, which is specifically designed for use in resource-limited settings [28]. In brief, sagittal images were obtained in six lung regions of each hemithorax, covering the anterior, lateral and dorsolateral areas of the chest. In children younger than twelve years, LUS was performed according to SOP adapted for a paediatric population [34]. The chest was divided into twelve lung regions: upper and lower; left and right; anterior (mid-clavicular line), posterior (mid-scapular line) and lateral (mid-axillary line). Scanning was performed in the longitudinal and transverse planes, moving the probe systematically from the sub-clavicular region to the diaphragm.

For the full IVC and LUS protocols, see Online Resource 1.

### US outcomes and interpretation

All US images were interpreted by the sonographer (CMP) and a second independent expert reader (MAH). Each reader reported on the overall quality of the images as ‘good’ (all views were clearly interpretable), ‘moderate’ (most, but not all, views were clearly interpretable) or ‘poor’ (most views were uninterpretable). Poor quality images were excluded. In the case of discrepancies in interpretation between the two readers, a third expert reader (CCCH) was consulted to arrive at a final decision. Second and third readers were blinded to clinical data and to each other’s findings.

#### Volume status measures

IVC-CI was calculated according to the following formula: ([max IVC–min IVC]/max IVC × 100%). IVC/Ao ratio was calculated by dividing the maximal A–P diameters of the IVC and aorta, respectively. Dehydration was defined as IVC-CI ≥ 50% or IVC/Ao ≤ 0.8 [35].

#### Lung aeration measures

Each lung region was assessed using a pre-defined lung aeration scoring system [28], of which the most abnormal score was recorded: (1) A pattern (i.e. normal aeration), defined as the presence of lung sliding with A-lines or no more than 2 isolated B-lines; (2) B1 pattern (i.e. moderate loss of lung aeration), defined as multiple (> 2) well-defined B-lines; (3) B2 pattern (i.e. severe loss of lung aeration), defined as multiple coalescent B-lines; and (4) C pattern (i.e. consolidation), defined as a subpleural, echo-poor or tissue-like area > 0.5 cm with or without air bronchograms and causing disruption of the pleural line. Additionally, subpleural nodules (defined as subpleural consolidations < 0.5 cm causing disruption of the pleural line) and pleural effusions (i.e. anechoic collections between the pleural line and chest wall) were separately noted.

#### Composite lung aeration measures

An interstitial syndrome was defined as having at least 2 lung regions showing a B pattern (i.e. either a B1 or B2 pattern). This was interpreted as evidence of reduced lung aeration and/or increased lung density, which occurs most commonly in the setting of pulmonary oedema. Other, albeit less common, causes of unilateral interstitial syndrome include interstitial pneumonia, tuberculosis, chronic bronchitis, acute bronchiolitis, atelectasis and tumours [27, 28].

### Data analysis

Baseline characteristics were analysed using counts and proportions for categorical variables and median and interquartile range for non-normally distributed continuous variables. Where appropriate, the data were log transformed. Proportions were compared with the Chi-square test or Fisher’s exact test, as appropriate. Medians were compared using Mann–Whitney *U* tests (2 groups) or Kruskal–Wallis tests (3 groups). Correlations were assessed with Spearman’s rank treating US variables as linear and using the Holm–Sidak method to correct for multiple hypothesis testing. Inter-rater agreement was evaluated using the Kappa Cohen coefficient (*K*). A *p* value of < 0.05 was considered statistically significant. All statistical analyses were performed using GraphPad Prism (version 8.4.3).

## Results

### Baseline characteristics

**Fig. 2:**
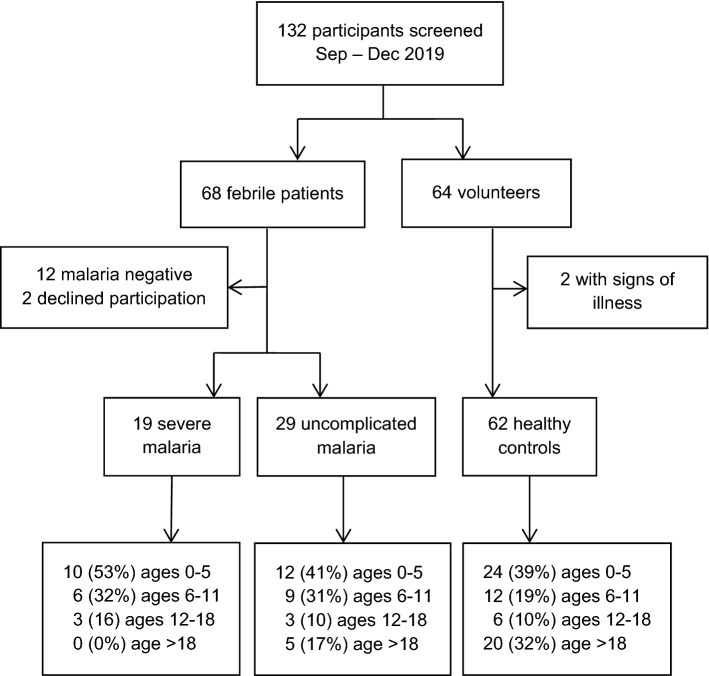
Screening and enrolment flow chart

A total of 132 individuals were screened for eligibility, including 68 febrile patients and 64 community volunteers. Of these, 110 were enrolled, including 48 patients with falciparum malaria and 62 healthy controls (Fig. 2).

Baseline demographics and clinical characteristics of the participants are presented in Table 1. Among malaria patients, 19 had strictly defined severe malaria, one of whom died and 29 had uncomplicated malaria, none of whom died. The age range in malaria patients was from 5 months to 75 years, with 77% (*n* = 37) being under 12 years. The healthy volunteer group was slightly older than both the severe and uncomplicated malaria groups. A recent history of cough or rhinitis was more frequent in malaria patients compared to healthy participants.

**Table 1:** Participant baseline characteristics

	Severe malaria (*n* = 19)	Uncomplicated malaria (*n* = 29)	Healthy participants (*n* = 62)	*p* (overall)	*p* (SM vs UM)
Age (years)	4.5 (1.8–7.6)	6.6 (3.0–12.6)	8.2 (3.8–28.5)	0.013	0.12
0–5 years	1.8 (1.4–2.7)	2.3 (1.6–3.4)	3.5 (2.5–4.7)	0.024	0.25
6–11 years	7.2 (6.7–7.7)	7.8 (6.5–9.5)	7.9 (6.9–9.0)	0.40	0.47
12–18 years	12.6 (12.3–12.9)	13.2 (12.1–14.3)	15.3 (13.4–18.2)	0.10	0.70
> 18 years	–	39.2 (25.1–70.4)	30.6 (28.5–35.6)	0.26	–
Female sex	12 (37)	19 (41)	27 (44)	0.87	> 0.99
WFL %ile, 0 to < 2 years^a^	47 (23–62)	26.1 (2–61)	41 (30–66)	0.47	0.33
BMI-for-age %ile, 2 to < 20 years^b^	16 (1–36)	16 (7–32)	23 (3–54)	0.47	0.71
BMI (kg/m^2^), ≥ 20 years^c^	–	26 (25–27)	25 (21–28)	0.58	–
Past medical history					
Anaemia	2 (11)	1 (3)	0 (0)	0.20	0.55
Allergic rhinitis	0 (0)	0 (0)	1 (2)	0.41	–
Asthma	2 (11)	0 (0)	1 (2)	0.95	0.15
Cardiac arrhythmia	1 (5)	0 (0)	0 (0)	0.73	0.40
History of severe malaria^d^	7/12 (58)	18/23 (78)	26/44 (59)	0.26	0.26
Hypertension	0 (0)	0 (0)	2 (3)	0.24	–
Obesity	0 (0)	0 (0)	2 (3)	0.24	–
Recent cough or rhinitis	8 (42)	15 (52)	16 (26)	0.044	0.57
Sickle cell disorders^e^	1 (5)	0 (0)	1 (2)	0.74	0.40
Tobacco use, current or former	0 (0)	1 (5)	1 (2)	0.62	> 0.99
Clinical findings					
Tympanic temperature (°C)	37.6 (37.5–38.2)	37.7 (36.8–38.7)	36.9 (36.8–37.1)	< 0.0001	0.66
Impaired consciousness	11 (58)	0 (0)	n/a	–	< 0.0001
Convulsions this illness	5 (26)	0 (0)	n/a	–	0.007
Jaundice visible to clinician	8 (42)	2 (7)	n/a	–	0.008
Sunken eyes	8 (42)	8 (28)	0 (0)	< 0.0001	0.36
Dry mucous membranes	7 (37)	0 (0)	1 (2)	< 0.0001	0.0008
Capillary refill time ≥ 2 s	12 (63)	9 (32)	0 (0)	< 0.0001	0.043
Weak pulse	2 (11)	2 (7)	0 (0)	0.018	> 0.99
Cold extremities	4 (21)	0 (0)	0 (0)	0.0002	0.022
Tachycardia	8 (42)	11 (38)	3 (5)	< 0.0001	> 0.99
Severe tachycardia	2 (11)	3 (10)	0 (0)	0.018	> 0.99
Tachypnoea	11 (58)	12 (41)	4 (7)	< 0.0001	0.38
SpO_2_ ≤ 94%	4 (21)	0 (0)	0 (0)	0.0003	0.024
Increased respiratory effort	12 (63)	7 (24)	0 (0)	< 0.0001	0.015
Crepitations on auscultation	5 (26)	3 (11)	0 (0)	< 0.0001	0.24
Laboratory findings^f^					
Haemoglobin, g/dL	5.1 (4.2–8.0)	9.7 (8.0–10.3)	n/a	–	0.0001
White cell count, × 10^3^/µL	12.7 (7.5–23.1)	6.0 (5.1–8.1)	n/a	–	< 0.0001
Platelets, × 10^3^/µL	253 (206–390)	148 (76–200)	n/a	–	0.0005
Parasite count, × 10^3^/µL^g^	33.0 (9.1–123)	11.1 (3.5–58.7)	n/a	–	0.16
Intravenous fluids in first 24 h, mL/kg^h^	50 (42–56)	46 (34–60)	n/a	–	0.35
Blood transfusion	11 (58)	3 (10)	n/a	–	0.0008

Shown is median (inter-quartile range, IQR) for nonbinary data or count (proportion) for binary data. Chi-square or Fisher’s exact test used for categorical variables, Mann–Whitney-*U* test or Kruskal–Wallis test for continuous variables. Overall *p* compares groups of healthy participants, severe and uncomplicated malaria

*BMI* body mass index; *SM* severe malaria; *SpO*_*2*_ oxygen concentration; *UM* uncomplicated malaria; *WFL* weight for length

^a^The data are for 6 (SM), 5 (UM) and 3 (control) participants

^b^The data are for 10 (SM), 18 (UM) and 37 (control) participants

^c^The data are for 4 (UM) and 20 (control) participants

^d^History of severe malaria was defined as having ≥ 1 prior episode of malaria requiring inpatient hospitalisation

^e^Sickle cell disorders included sickle cell disease (*n* = 1, malaria group) and sickle cell trait (*n* = 1, control group)

^f^The data are for 18 (SM) and 27 (UM) participants

^g^The data are for 9 (SM) and 20 (UM) participants

^h^The data are for 16 (SM) and 25 (UM) participants

### US investigation

Baseline IVC and LUS examinations were performed in 105 (95%) and 108 (98%) participants, respectively. Combining all time points (days 0, 1, 2 and 3), 192 serial IVC US studies and 198 serial LUS studies were completed, with the latter amounting to 2298 scanned lung regions. Of the completed US studies, 100% and 99% of IVC and LUS scans, respectively, were considered interpretable and therefore included in the analysis. The remaining one percent of LUS scans was not interpretable due to either the heart obstructing the lung fields or poor image quality secondary to poor participant compliance. Participant compliance was considered good in 78% of cases, moderate in 10% of cases and poor in 12% of cases. In 92% of assessments, participants were scanned in the supine position; the remaining assessments were performed with participants seated in the caregiver’s lap (all children ≤ 3 years of age). Median time to perform a full US examination, including IVC and LUS components, was 8 min (IQR: 7, 10).

There was substantial inter-rater agreement between the sonographer and second reader for normal lung aeration (A pattern) (*K* = 0.70), more than two B-lines in one intercostal space (B pattern) (*K* = 0.67), consolidation (C pattern) (*K* = 0.70) and pleural effusion (*K* = 0.71).

### Assessment of hypovolaemia

Eighty-four percent (*n* = 16) of severe malaria patients had more than one feature of dehydration or impaired perfusion on enrolment compared with 41% (*n* = 12) of uncomplicated malaria patients and none of the controls (*p* < 0.0001). Two patients with severe malaria (11%) had hypotension according to WHO severity criteria. Other clinical signs of hypovolaemia in the different categories of participants are shown in Table 1. Of note, no participants had clinical evidence of reduced abdominal skin turgor in this study.

**Fig. 3:**
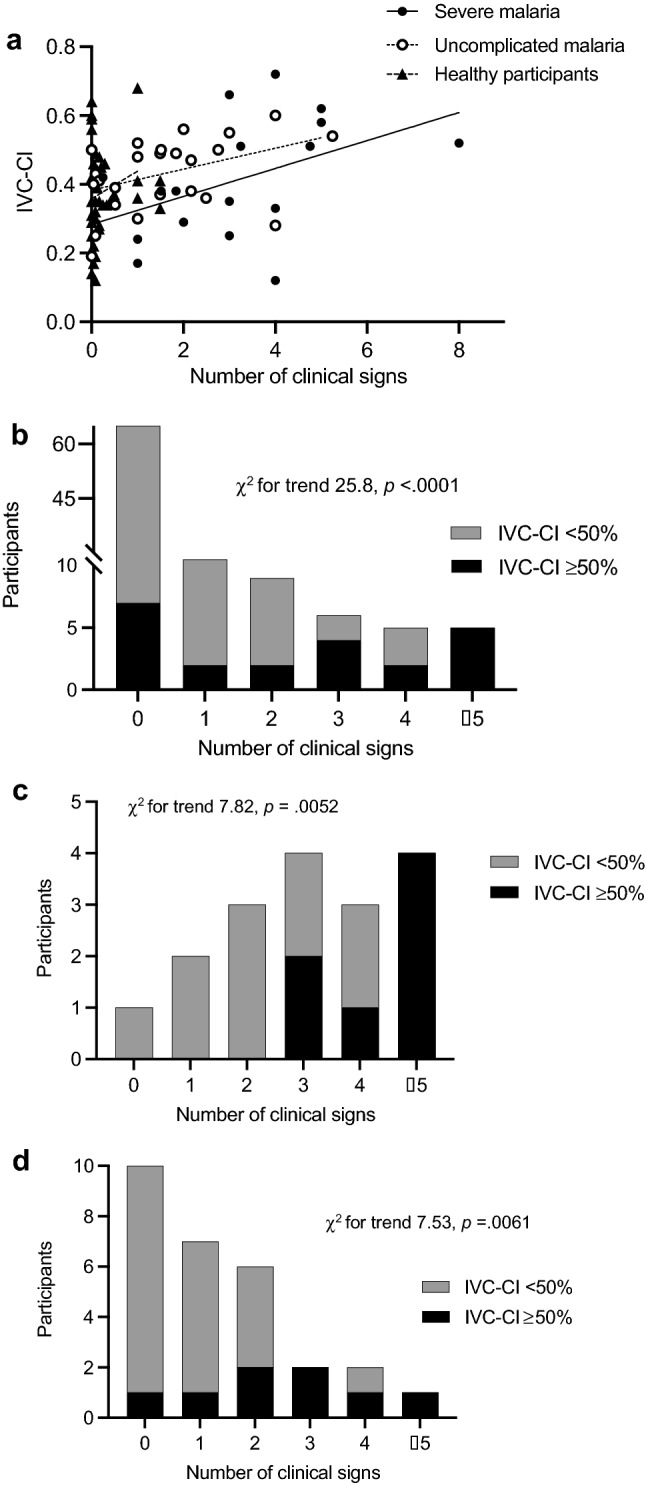
Relationship between IVC collapsibility and frequency of clinical signs of hypovolaemia on enrolment. The data shown are for all participants, *n* = 104 (**a**, **b**); patients with severe malaria, *n* = 17 (**c**); and patients with uncomplicated malaria, *n* = 28 (**d**). *IVC-CI* inferior vena cava collapsibility index. Clinical signs included sunken eyes, dry mucous membranes, prolonged capillary refill time, weak peripheral pulse, cold extremities, severe tachycardia, hypotension, increased respiratory effort and/or tachypnoea as defined in Methods section

An elevated IVC-CI on enrolment reflecting intravascular fluid depletion was associated with an increased number of clinical signs of hypovolaemia in patients with severe (*r* = 0.50, *p* = 0.022*, *n* = 17) and uncomplicated malaria (*r* = 0.44, *p* = 0.0096*, *n* = 28), but not in healthy participants (*r* = 0.14, *p* = 0.14, *n* = 59; *denotes correlation that remained statistically significant after the Holm–Sidak correction) (Fig. 3). Likewise, in each age sub-group, participants with an IVC-CI ≥ 50% on enrolment had a significantly greater number of clinical signs of hypovolaemia (Table 2).

**Table 2:** Relationship between IVC US measures and frequency of clinical signs of hypovolaemia on enrolment by age sub-groups

	Age group (years)	Number of clinical signs^a^						${x}^{2}$ for trend	*p*
		0	1	2	3	4	≥ 5		
IVC-CI ≥ 50%	All	7/65 (11)	2/14 (14)	2/9 (22)	4/6 (67)	2/5 (40)	5/5 (100)	25.8	< 0.0001
	0–5	5/20	0/7	2/6	1/2	2/4	2/2	4.92	0.027
	6–11	1/18	0/4	0/1	2/2	0/1	1/1	9.73	0.0018
	12–18	0/5	0/1	0/2	0/1	–	2/2	6.52	0.011
	> 18	1/22	2/2	–	1/1	–	–	15.7	< 0.0001
IVC/Ao ≤ 0.8	All	8/65 (12)	3/14 (21)	1/9 (11)	1/6 (17)	0/5 (0)	2/5 (40)	0.67	0.41
	0–5	3/20	0/7	1/6	0/2	0/4	0/2	1.01	0.31
	6–11	4/18	2/4	0/1	0/2	0/1	0/1	0.81	0.37
	12–18	0/5	0/1	0/2	1/1	–	2/2	8.31	0.0039
	> 18	1/22	1/2	–	0/1	–	–	1.17	0.28

The data are presented as counts (proportions)

^a^Clinical signs include sunken eyes, dry mucous membranes, prolonged capillary refill time, weak peripheral pulse, cold extremities, severe tachycardia for age, hypotension for age, increased respiratory effort and/or tachypnoea for age, as defined in Methods

Among patients with severe malaria, a low IVC/Ao ratio on enrolment reflecting a low-volume state was associated with an increased number of clinical signs of hypovolaemia (*r* = –0.45, *p* = 0.034, *n* = 17); however, this relationship was not observed in the uncomplicated malaria (*r* = 0.23, *p* = 0.12, *n* = 28) or control groups (*r* = 0.04, *p* = 0.38, *n* = 59). Participants aged 12–18 years who had an IVC/Ao ratio ≤ 0.8 on enrolment tended to have more clinical signs of hypovolaemia (*p* = 0.0039), but this trend was not observed in other age sub-groups (Table 2).

Table 3 illustrates the relationship between IVC US parameters and individual clinical signs of hypovolaemia on enrolment in all participants. Notably, we found strong evidence of a higher IVC-CI in participants with sunken eyes as compared to those without sunken eyes (*p* = 0.0001*) and in participants with CRT ≥ 2 s as compared to those with CRT < 2 s (*p* = 0.001*) (Table 3). In contrast, a low IVC/Ao ratio did not correlate significantly with any clinical signs of hypovolaemia on enrolment (Table 3).

**Table 3:** Relationship between IVC US measures and clinical signs of hypovolaemia on enrolment in all participants

Clinical finding		*N*	IVC-CI					IVC/Ao		
			≥ 50%, *n* (%)	*p*	rho	*p*	≤ 0.8, *n* (%)	*p*	rho	*p*
Eyes	Sunken	14	8 (57)	0.002	0.35	0.0001*	3 (21)	0.33	0.002	0.49
	Normal	90	14 (16)				12 (13)			
Mucous membranes	Dry	7	3 (43)	0.16	−0.005	0.48	2 (29)	0.27	−0.07	0.23
	Normal	97	19 (20)				13 (13)			
CRT	Prolonged	20	11 (55)	0.0002	0.29	0.001*	4 (20)	0.32	−0.07	0.24
	Normal	84	11 (13)				11 (13)			
Peripheral pulse	Weak	4	3 (75)	0.029	0.16	0.049	1 (25)	0.47	−0.06	0.29
	Normal	100	19 (19)				14 (14)			
Extremities	Cold	4	3 (75)	0.029	0.21	0.016	0 (0)	0.53	0.003	0.49
	Warm	100	19 (19)				15 (15)			
HR	Severe tachycardia^a^	5	4 (80)	0.007	0.19	0.025	2 (40)	0.15	−0.13	0.089
	No tachycardia	82	11 (13)				13 (13)			
SBP	Hypotension^a^	2	2 (100)	0.042	0.22	0.020	1 (50)	0.27	−0.09	0.20
	No hypotension	84	16 (19)				12 (14)			
Respiratory effort	Increased	18	10 (56)	< 0.0001	0.15	0.069	4 (22)	0.24	−0.09	0.17
	Normal	86	12 (14)				11 (13)			
RR	Tachypnoea^a^	24	10 (42)	0.008	0.17	0.042	4 (17)	0.47	−0.12	0.12
	No tachypnoea	80	12 (15)				11 (14)			

Chi-square test or Fisher’s exact test for categorical variables. Spearman’s rank for correlation coefficients

^*^Indicates Spearman correlation that remained statistically significant after the Holm–Sidak correction

*CRT* capillary refill time; *HR* heart rate; *IVC-CI* inferior vena cava collapsibility index; *IVC/Ao* inferior vena cava-to-aorta ratio; *rho* Spearman’s correlation coefficient; *RR* respiratory rate; *SBP* systolic blood pressure

^a^As defined in the Methods section

When measured concurrently with clinical signs on days 1–3 in malaria patients, a high IVC-CI correlated with moderate tachycardia (*r* = 0.26, *p* = 0.009*, *n* = 85) and increased respiratory effort (*r* = 0.25, *p* = 0.011*, *n* = 86), but not with sunken eyes, prolonged CRT or tachypnoea (Online Resource 2). Other clinical signs on days 1 through 3 were either not observed or occurred in less than 2% of assessments. Similarly, a low IVC/Ao ratio on days 1 through 3 in malaria patients correlated with moderate tachycardia (*r* = –0.30, *p* = 0.003*, *n* = 85) and increased respiratory effort (*r* = –0.37, *p* = 0.0003*, *n* = 86), but not with other observed signs of hypovolaemia (Online Resource 2).

### Assessment of pulmonary oedema

Among patients with severe malaria (*n* = 19), three (16%) presented with clinical signs suggestive of pulmonary oedema on admission (SpO2 < 94% and RR > 30/min). Of these, one patient had bilateral crepitations on auscultation and evidence of both a B and C pattern on LUS (Box 1, Clinical case vignette II). The remaining two patients had clear lungs on auscultation; one had a B pattern on LUS and the other had normal lung aeration in all fields. In the control group, all participants had normal respiratory findings with exception of four children (aged 3–7 years) with mild tachypnoea.

**Fig. 4:**
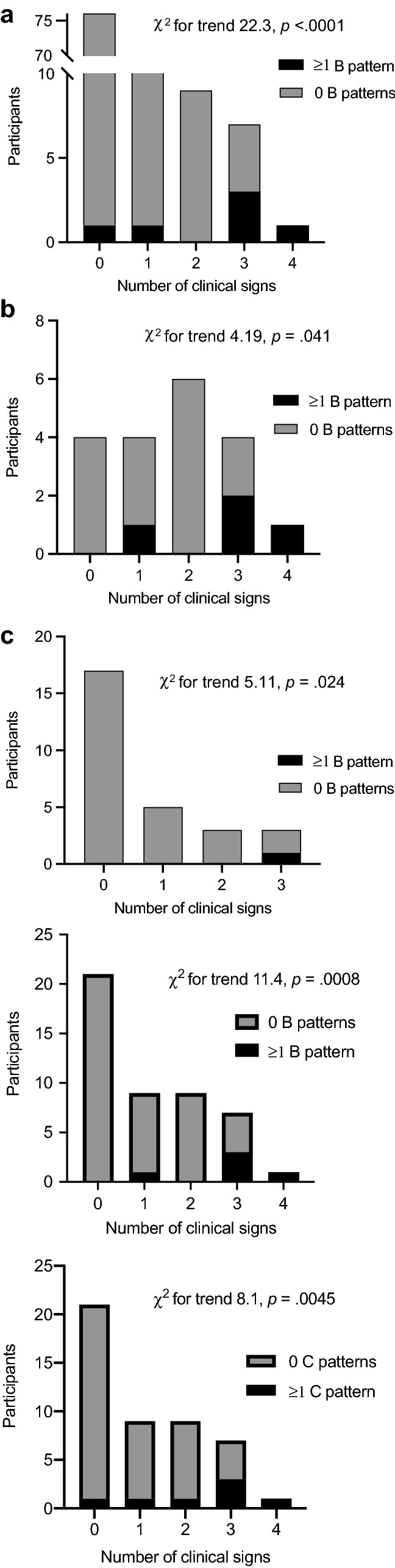
Relationship between a B pattern on LUS and frequency of clinical signs associated with pulmonary oedema on enrolment. The data shown are for all participants, *n* = 106 (**a**); patients with severe malaria, *n* = 19 (**b**); and patients with uncomplicated malaria, *n* = 28 (**c**). Clinical signs included increased respiratory effort, crepitations on lung auscultation, tachypnoea and/or oxygen saturation ≤ 94% as defined in Methods section

The number of lung fields showing a B pattern on enrolment reflecting interstitial fluid was associated with an increased number of clinical signs of pulmonary oedema in severe (*r* = 0.45, *p* = 0.027, *n* = 19) and uncomplicated malaria patients (*r* = 0.34, *p* = 0.039, *n* = 28), but not in healthy participants (*r* = –0.03, *p* = 0.40, *n* = 59) (Fig. 4). In each age sub-group, participants who had ≥ 1 B pattern on enrolment had significantly more clinical signs of pulmonary oedema (Table 4). By contrast, the number of lung fields showing a C pattern on enrolment reflecting a possible chest infection did not correlate significantly with the number of clinical signs of pulmonary oedema in any group (severe malaria, *r* = 0.23, *p* = 0.17, *n* = 19; uncomplicated malaria, *r* = 0.12, *p* = 0.26, *n* = 28; healthy, *r* = –0.06, *p* = 0.32, *n* = 59).

**Table 4:** Relationship between abnormal LUS patterns and frequency of clinical signs of pulmonary oedema on enrolment by age sub-groups

	Age group (years)	Number of clinical signs^a^				${x}^{2}$ for trend	*p*
		0	1	2	≥ 3		
≥ 1 B pattern	All	1/76 (1)	1/13 (8)	0/9 (0)	4/8 (50)	19.8	< 0.0001
	0–5	0/21	1/10	0/6	2/5	6.17	0.013
	6–11	0/23	0/1	0/2	1/1	13.1	0.0003
	12–18	0/7	0/2	0/1	1/2	3.92	0.048
	> 18	1/25	–	–	–	–	–
≥ 1 C pattern	All	4/76 (5)	1/13 (8)	1/9 (11)	3/8 (38)	7.48	0.0062
	0–5	0/21	1/10	0/6	3/5	10.5	0.0012
	6–11	0/23	0/1	1/2	0/1	5.21	0.023
	12–18	2/7	0/2	0/1	0/2	1.28	0.26
	> 18	2/25	–	–	–	–	–

The data are presented as counts (proportions)

^a^Clinical signs include increased respiratory effort, crepitations, oxygen saturation ≤ 94% and/or tachypnoea for age, as defined in Methods

Table 5 illustrates the relationship between LUS findings and individual clinical signs associated with pulmonary oedema on enrolment in all participants. Notably, we found no evidence of a difference in the number of B patterns on enrolment in participants with and without tachypnoea (*p* = 0.069), but strong evidence of an increased number of B patterns in participants with increased respiratory effort versus normal respiration (*p* = 0.0003*), crepitations versus clear lungs on auscultation (*p* < 0.0001*) and SpO_2_ ≤ 94% versus SpO_2_ > 94% (*p* < 0.0001*) (Table 5).

**Table 5:** Relationship between LUS abnormalities and clinical signs of pulmonary oedema on enrolment in all participants

Clinical finding		*N*	B pattern				C pattern			
			≥ 1 area, *n* (%)	*p*	# of areas, rho	*p*	≥ 1 area, *n* (%)	*p*	# of areas, rho	*p*
Respiratory effort	Increased	18	4 (22)	0.007	0.32	0.0003*	5 (28)	0.007	0.31	0.0006*
	Normal	88	2 (2)				4 (5)			
Lung auscultation	Crepitations	8	4 (50)	0.0002	0.55	< 0.0001*	4 (50)	0.002	0.42	< 0.0001*
	Normal	98	2 (2)				5 (5)			
RR	Tachypnoea^a^	26	3 (12)	0.16	0.15	0.069	2 (8)	0.61	–0.02	0.42
	Normal	80	3 (4)				7 (9)			
SpO_2_	≤ 94%	4	3 (75)	0.0004	0.59	< 0.0001*	1 (25)	0.30	0.11	0.12
	> 94%	101	3 (3)				8 (8)			

Chi-square test or Fisher’s exact test for categorical variables. Spearman’s rank for correlation coefficients

*rho* Spearman’s correlation coefficient; *RR* respiratory rate; *SpO*_*2*_ oxygen saturation

^*^Indicates Spearman correlation that remained statistically significant after the Holm–Sidak correction

^a^As defined in Methods section

When assessed on days 1–3 in malaria patients, the number of lung fields showing a B pattern correlated only with increased respiratory effort (*r* = 0.23, *p* = 0.013, *n* = 89) (Online Resource 2).

### Markers of clinical severity

Median (IQR) IVC-CI on enrolment was increased in malaria patients (42% [34–51]) relative to healthy participants (35% [29–45]; *p* = 0.01), but was similar between patients with severe (38% [27–55]) and uncomplicated malaria (44% [36–50]; *p* = 0.055). The share of participants with IVC-CI ≥ 50% on enrolment increased significantly from healthy participants to uncomplicated to severe malaria (test for trend *p* < 0.0001) (Table 6).

**Table 6:** Relationship between high IVC-CI and low IVC/Ao ratio and malaria vs. no malaria on enrolment

	Severe malaria	Uncomplicated malaria	Healthy participants	${x}^{2}$ for trend	*p*
IVC-CI ≥ 50%	7/17 (41)	8/28 (29)	7/59 (12)	46.5	< 0.0001
IVC/Ao ≤ 0.8	3/17 (18)	5/28 (18)	6/59 (10)	1.0	0.31

*IVC-CI* inferior vena cava collapsibility index; *IVC/Ao* inferior vena cava-to-aorta ratio

Median (IQR) IVC/Ao ratio on enrolment was lower in malaria patients (0.93 [0.83–1.07]) compared to healthy participants (1.03 [0.92–1.16]; *p* = 0.014), but did not differ significantly between the severe (0.97 [0.54–1.09]) and uncomplicated malaria groups (0.92 [0.82–1.04]; *p* = 0.36). There was no significant trend from healthy participants to uncomplicated to severe malaria in terms of the share of participants with an IVC/Ao ratio ≤ 0.8 on enrolment (test for trend *p* = 0.31) (Table 6).

Table 7 presents LUS findings on enrolment stratified by study group. Notably, a B pattern was observed more frequently from healthy participants to uncomplicated to severe malaria (test for trend *p* = 0.004).

**Table 7:** Relationship between frequency of abnormal LUS patterns on enrolment and study groups

	Severe malaria	Uncomplicated malaria	Healthy participants	${x}^{2}$ for trend	*p*
≥ 1 B pattern	4/19 (21)	1/28 (4)	1/59 (2)	8.25	0.004
≥ 1 C pattern	4/19 (21)	2/28 (7)	3/59 (5)	3.95	0.047
≥ 1 LUS abnormality^a^	7/19 (37)	2/28 (7)	4/59 (7)	9.23	0.002

*LUS* lung ultrasound

^a^Includes the number of lung fields showing a B pattern, C pattern or pleural effusion

**Fig. 5:**
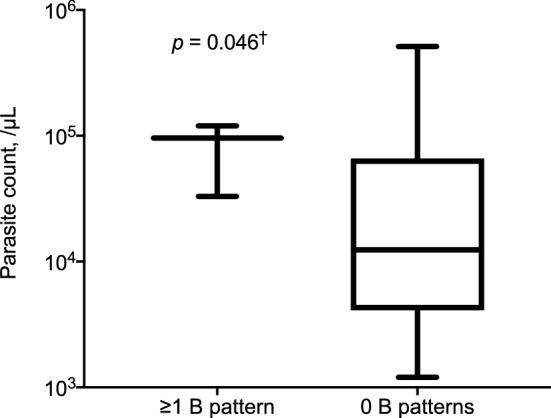
Median parasite count and number of lung fields showing a B pattern on enrolment in 29 malaria patients. Boxes represent median (IQR); whiskers represent range. ^†^Not statistically significant after the Holm–Sidak correction

Among malaria patients, an increased number of lung fields showing a B-pattern on enrolment correlated significantly with parasite count (*r* = 0.32, *p* = 0.046, *n* = 29). Patients with ≥ 1 B pattern on enrolment had a median (IQR) parasite count of 96.0 × 10^3^/µL (33.0–120) compared to 12.4 × 10^3^/µL (6.0–54.6) in those without a B pattern (*p* = 0.046; Fig. 5). No correlations were found between parasite count and IVC-CI or IVC/Ao ratio.

### Relationship between IVC and LUS

**Fig. 6:**
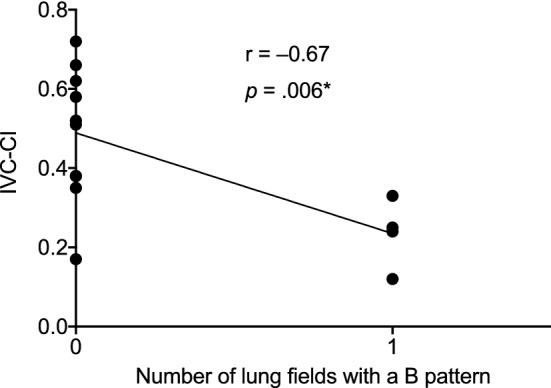
Relationship between IVC collapsibility and number of lung fields showing a B pattern on enrolment in severe malaria. *Remained statistically significant after the Holm–Sidak correction

A low IVC-CI on enrolment reflecting possible volume overload was significantly associated with the number of lung fields showing a B-pattern in patients with severe malaria (*p* = 0.006*; Fig. 6), but not in the uncomplicated malaria (*r* = –0.15, *p* = 0.24) or control groups (*r* = 0.06, *p* = 0.34). Median (IQR) IVC-CI in severe malaria patients with and without a B pattern on enrolment was 25% (15–31) and 52% (7–63), respectively (*p* = 0.006), although this relationship was not observed at 24 or 48 h.

No significant associations were observed between IVC/Ao ratio and the number of lung fields showing a B pattern in any study group.

### US measures and fluid administration

**Fig. 7:**
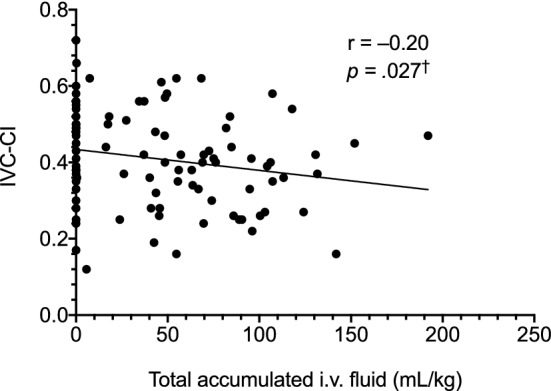
Relationship between IVC collapsibility and accumulated total intravenous fluid until the point of measurement at 0, 24 and 48 h in 43 malaria patients. The data shown are for 91 separate time points where both fluid volume and ultrasound data were available. ^†^Not statistically significant after the Holm–Sidak correction

Total accumulated intravenous fluid (mL/kg) administered until the point of US measurement at 0-, 24- and/or 48-h time points was available for 43 malaria patients. fluid IVC-CI tended to be lower in patients with a higher total accumulated fluid volume (*p* = 0.027; Fig. 7). No correlations were found between the total accumulated fluid volume and either IVC/Ao or the number of lung fields showing a B pattern on enrolment.

### Clinical case vignettes

Three concise case reports illustrate practical examples (Box 1).

Box 1 Clinical case vignettes **Clinical case vignette I**

A 5-year-old male child presented with severe falciparum malaria. On physical examination, oral temperature was 38.2 °C, HR 122 bpm, SBP 100 mmHg, RR 44 breaths/min, SpO_2_ 97%. He was restless and irritable. Eyes appeared sunken and jaundiced. Respirations were deep and laboured, and the lungs were clear to auscultation bilaterally. Palpation of the bilateral lower extremities revealed rapid pulses and warm/dry skin. Capillary refill was > 2 s. Labs on admission showed WBC 5.5, platelets 156, haemoglobin 11.0 g/dL, haematocrit 32.9% and parasite count of 480,000/µL. Baseline US examination showed IVC-CI 54%, IVC/Ao 1.21 and normal lung aeration in all fields. Intravenous fluids were started at a rate of 2.6 cc/kg/h.

Vital parameters normalised over the next day, but the RR remained elevated at 36 breaths/min on Days 1 and 2; and then at 32 on Day 3. IVC-CI declined to around 40%, IVC/Ao decreased to 1.0 on Day 1, then stabilized at around 1.3 on days 2 and 3. Lung aeration remained normal throughout the hospital course.

*Summary:* This patient had evidence of dehydration/impaired perfusion on examination, including rapid/deep respirations with clear lungs on auscultation. Baseline US examination showed IVC-CI of 54% and normal lung aeration. Taken together, both clinical and US examination findings supported a diagnosis of hypovolaemia. This was further confirmed by the patient’s improved clinical status and IVC-CI of 40% following fluid administration.

**Clinical case vignette II**

An 18-month-old female presented with severe falciparum malaria complicated by 4 days of non-bloody diarrhoea and vomiting and impaired consciousness. Baseline vitals were notable for a temperature of 38.1 °C, SBP 80 mmHg, HR 141 bpm, RR 34 breaths/min and SpO_2_ 93%. On physical examination, the patient was lethargic with severe prostration. Respirations were deep and laboured with evidence of chest indrawing and scattered crepitations on lung auscultation. Grade II splenomegaly was noted. Palpation of the bilateral lower extremities revealed rapid pulses and cool skin. Capillary refill time was > 2 s. Laboratory analyses were as follows: WBC 23.5, platelets 146, haemoglobin 4.6 g/dL, haematocrit 13.5%, glucose 11.16 mmol/L and a parasite count of 33,000/µL. Baseline IVC-CI was 33% and IVC/Ao was 0.81. LUS was notable for B patterns in the left posterior lung fields and a left lateral pleural effusion with fibrinous exudate. Treatment with intravenous artesunate was initiated. At the discretion of the treating clinician, intravenous fluids were held due to suspicion for pulmonary oedema.

On Day 1, the patient remained lethargic and prostrated. Vitals were 36.6 °C, HR 115 bpm, RR 44 breaths/min, SpO_2_ 99%. Lungs were clear to auscultation bilaterally and the patient was breathing comfortably on room air. Capillary refill and peripheral pulses were normal. US examination showed IVC-CI 28%, IVC/Ao 1.09 and normal lung aeration in the anterior and lateral lung fields bilaterally. Posterior lung fields were not assessed, per the caregiver’s request to maintain patient comfort. Fluid therapy was later initiated at a rate of 2 cc/kg/h and the patient received a blood transfusion for severe malarial anaemia (240 cc whole blood administered over 4 h).

On Day 2, the patient was fully alert. Vitals were 38.0 °C, HR 134 bpm, RR 36 breaths/min, SpO_2_ 99%. Mild crepitations were apparent on lung auscultation. The physical examination was otherwise unremarkable. US scanning yielded IVC-CI 27% and IVC/Ao 1.21. LUS showed a mixture of B and C patterns in the right lateral fields (subpleural consolidations and coalescent B lines) and a left lateral pleural effusion with fibrinous exudate and collapsed lung tissue.

On Day 3, vitals were 38.1 °C, SBP 76 mmHg, HR 131 bpm, RR 32 breaths/min and SpO_2_ 96%. Physical examination was unremarkable. IVC-CI and IVC/Ao were 30% and 1.18, respectively. LUS showed a mixture of B and C patterns in the right posterior fields and subpleural consolidation in the right lateral field.

*Summary:* This patient with severe malaria presented with severe respiratory distress on admission with associated crepitations and oxygen saturation of 93%. LUS showed evidence of a B pattern in more than one lung field concerning for pulmonary oedema or possibly bacterial pneumonia given that the WBC count was elevated. Intravenous fluids were therefore held for the first 24 h of admission, then started at a rate of 2 cc/kg/h. Lung examination and LUS improved initially on Day 1, but then showed evidence of worsening on Days 2 and 3, with recurrence of mild crepitations and a mixture of B and C patterns in more than one lung field. IVC collapsibility remained around 30% and IVC/Ao increased from 0.81 at baseline to a peak of 1.21 on Day 2, suggesting possible fluid overload.

**Clinical case vignette III**

A 12-year-old male presented with severe falciparum malaria complicated by impaired consciousness, convulsions and prostration. The patient was admitted overnight and enrolled in the study the following morning. Prior to enrolment, iv fluids had been initiated at a rate of 1.5 cc/kg/h and the patient had already received an estimated 500 cc of fluid and a blood transfusion (450 cc) at study baseline. On examination, temperature was 37.5 °C, HR 115 bpm, SBP 98 mmHg, RR 56 breaths/min, SpO_2_ 99%. He was alert and oriented. Eyes were jaundiced. Increased respiratory effort and bilateral crepitations on lung auscultation were noted. Severe hepatosplenomegaly was also noted. Capillary refill and peripheries were normal. Labs on admission showed WBC 7.2, platelets 233, haemoglobin 3 g/dL, haematocrit 8% and parasite count of 120,000/µL. Baseline US examination showed IVC-CI 25%, IVC/Ao 0.8, a B pattern in the right lower posterior lung fields and trace posterior pleural effusions bilaterally.

Heart rate improved and was normal by Day 2. Over the hospital course, the patient continued to show mildly increased respiratory effort on examination with mild crepitations. RR decreased, but remained elevated (52, 42, 40 on Days 1, 2 and 3, respectively). Peripheral oxygen saturation remained normal. IVC-CI increased and stabilised to around 40%. IVC/Ao remained less than 0.8. Number of lung fields with a B or C pattern increased to 2 on Days 1 and 2 and then increased to 5 on Day 3.

*Summary:* This patient with severe malaria received bolus fluids on admission and later presented with clinical signs of respiratory distress and reduced lung aeration on LUS. Additionally, IVC collapsibility was low, suggesting possible fluid overload. LUS findings continued to worsen over the hospital course.

## Discussion

Fluid overload leading to distinct features assessable by pulmonary ultrasound is not specific to severe malaria and to that end, the severe malaria pattern of ultrasound is neither disease-specific nor unique.

This exploratory study demonstrates the potential benefit of POCUS of the IVC and lungs for evaluating intravascular volume status and early signs of pulmonary oedema in malaria patients. Further prospective studies in various settings are needed to further explore the full potential of this application.

We observed that an increased vena cava collapsibility on enrolment, which is reflective of intravascular fluid depletion, correlated with most clinical signs of hypovolaemia, with exception of dry mucous membranes, highlighting the limited utility of this clinical sign in adults and children [14, 16, 18]. In addition, malaria patients with a high IVC-CI on enrolment tended to have an increased number of clinical signs of hypovolaemia. On the baseline LUS assessment, we observed that a B pattern, reflecting the presence of interstitial fluid, correlated well with clinical signs that can be associated with pulmonary oedema or a chest infection, including respiratory distress, crepitations on auscultation and reduced oxygen saturation. Among malaria patients, the presence of one or more LUS abnormalities on enrolment was associated with an increased number of clinical signs that can be associated with pulmonary oedema. This relationship was notably stronger among patients with a B-pattern, which is consistent with interstitial oedema, compared to those with a C-pattern, a sign of an infection.

Interestingly, neither a B- nor a C-pattern on enrolment correlated with tachypnoea, whereas increased IVC collapsibility was weakly associated with tachypnoea. These findings point to the challenges as well as the potential role of POCUS in helping to distinguish at the bedside between various causes of increased respiratory rate in malaria patients [36]. In a patient with tachypnoea for example, a highly collapsible IVC might help point clinicians towards a diagnosis of acidotic breathing, a physiologic compensatory response to acidosis, while multiple B lines might instead suggest underlying pulmonary oedema or chest infection. Taken together, these findings highlight the potential utility of POCUS for improved assessment of volume status as well as early detection of pulmonary oedema in malaria patients.

We observed that an IVC-CI ≥ 50% on enrolment indicating hypovolaemia increased from healthy participants to uncomplicated to severe malaria. A similar trend was also observed for abnormal LUS findings on enrolment, particularly the presence of a B pattern, which is consistent with interstitial oedema. In line with these findings, previous studies have demonstrated a relationship between IVC and LUS indices and clinical outcomes in malaria patients. In adults with malaria in India and Bangladesh, Kingston et al. found that fatal cases were associated with features of hypovolaemia, including increased IVC collapsibility and reduced cardiac index reserve [8]. Yacoub et al. similarly showed in Kenyan children with severe malaria that acidotic patients had more collapsible IVCs as well as other markers of hypovolaemia and mild cardiac dysfunction [7]. In the first study of its kind, Leopold and colleagues found a close association between the number of LUS abnormalities on admission and case fatality rates among Bangladeshi adults with malaria and sepsis. Furthermore, patients who developed ARDS over the course of the study had evidence of interstitial oedema on admission [28]. Thus, our findings lend further support to the hypothesis that POCUS may prove useful for risk stratification in malaria through early identification of hypovolaemia and pulmonary oedema.

Consistent with our hypothesis, we found that in patients with severe malaria, a B pattern on enrolment was inversely associated with IVC collapsibility. Additionally, an increased total accumulated intravenous fluid volume in malaria patients was modestly associated with a low IVC collapsibility. This suggests that in patients with suspected pulmonary oedema, a limited IVC collapsibility could serve as a helpful adjunct to clinical judgement for guiding fluid therapy in these patients.

It should be noted that the utility of IVC-CI as a surrogate measure of volume status remains subject of debate in the literature. Given its high compliance, the size and dynamics of the IVC vary with CVP, which in the absence of caval obstruction, are synonymous with RAP. Normal inspiration produces a drop in intra-thoracic pressure resulting in increased forward flow into the right atrium and a consequent decrease in IVC diameter. Elevation in CVP, such as with expiration or positive fluid balance, blunts forward caval flow and increases IVC diameter. The gold standard method for evaluating RAP involves invasive monitoring with placement of a central venous catheter; however, US represents a non-invasive, more widely accessible alternative [37]. Multiple studies have demonstrated a good correlation between IVC collapsibility and RAP (–0.50 < *r* ≤ –0.76) [38–41], although others have shown only modest associations [42]. There are also few studies conducted in children in the literature, with conflicting results [21, 43–45]. The diagnostic accuracy of IVC-CI is improved with the use of pre-defined cutoff values classifying RAP as either high or low [19, 37]. Based on these findings, the American Society of Echocardiography recommends using IVC-CI to estimate RAP in a variety of critical care settings, including patients with suspected hypovolaemia [46]. However, it remains unclear, whether IVC-CI can reliably predict fluid responsiveness in hypovolaemic patients. Two recent meta-analyses concluded that IVC-CI performs only moderately well in predicting fluid responsiveness; however, both reviews reported a high degree of heterogeneity between studies [46–48].

In this study, the IVC/Ao ratio did not correlate significantly with any baseline clinical signs of hypovolaemia in either malaria patients or healthy controls, although a low IVC/Ao ratio on enrolment was associated with an increased number of clinical signs of hypovolaemia in patients with severe malaria. Our results differ from those of previous studies in North American children showing that an IVC/Ao ratio ≤ 0.8 modestly predicts severe dehydration after acute diarrhoea [24, 35]. However, using the same cutoff value among rural children in Panama, Mazza et al. found no difference in IVC/Ao ratio between children with and without severe dehydration [49]. Two additional paediatric studies, one conducted in Rwanda [50], the other in Bangladesh [51], found a significant correlation between the aorta-to-IVC ratio and dehydration in children with acute diarrhoea; the larger study from Bangladesh concluded, however, that a cutoff value ≥ 2.0 was not sufficiently accurate to recommend as a stand-alone screening tool for dehydration in children [51]. In our study, malaria patients were younger. Post hoc analyses demonstrated that IVC/Ao was inversely associated with age, height and weight in malaria patients; whereas in controls, IVC/Ao correlated neither with age nor weight, but was positively correlated with height. Thus, it is likely that differences in age and body composition had at least some effect on our results. It is also possible that an IVC/Ao cutoff value of ≤ 0.8 previously validated in large, urban hospitals in the United States is not applicable to small, rural hospitals in low- and middle-income countries.

This study has several limitations. First, the sample size was relatively small, particularly the number of adult patients, all of whom had uncomplicated malaria. This reflects the typical epidemiology of malaria in areas of high endemicity, where development of partial immunity with time shifts the burden of cases primarily to children under five years of age [3]. Likewise, pulmonary manifestations were uncommon in our study sample, reflecting the fact that pulmonary oedema occurs rarely in children with malaria compared to adults [11]. Second, given the absence of a reference standard for evaluating intravascular volume status, we used physical examination findings in this study. However, clinical signs have been shown to correlate variably with fluid status. Third, while age matching should theoretically control for any impact of age on our results, age matching was imperfect in our study, with the control group being slightly older and the age range in malaria patients being much wider (Table 1). We therefore cannot exclude the possibility that imperfect age matching might have influenced our results. However, any impact of age is probably limited given that similar trends were observed when the data were stratified by age group (Tables 2, 4). Finally, patients in this study were not excluded if they had a concurrent diagnosis in addition to malaria. In contrast with the study in adults in Bangladesh [27], who [28], which did not find any C-pattern in patients with malaria, we found a C-pattern in six patients (12.5%), which is a finding specific for an infection. All six cases were among children, with half being ≤ 2 years of age (remaining patients were ages 3, 6 and 13 years). Concomitant bacterial infection is common in children with malaria [11]; thus, it is likely that at least some clinical and US findings were caused by other illnesses, such as pneumonia or gastroenteritis. To our knowledge, however, only two of the six patients with a C-pattern in our study had a documented co-infection and received antibiotics in addition to antimalarials: one was a 3-year-old male with uncomplicated malaria and acute bronchiolitis, the other a 2-year-old female with severe malaria and shigellosis. All six patients recovered quickly and survived to hospital discharge. Of note, our less restrictive inclusion criteria may increase the generalisability of our results to similar resource-limited healthcare settings, where diagnostic imaging and laboratory tests are often lacking.

## Conclusion

The majority of deaths in malaria patients occur in the first 24 h of admission. Thus, innovations to help improve management in this initial timeframe have the potential to improve outcomes substantially. Optimal fluid balance, in particular, is critically important to the management of malaria patients given the risks associated with intravascular fluid depletion and pulmonary oedema. In resource-constrained health systems, clinicians often have only the physical examination to guide their evaluation of volume status and fluid management in malaria patients. In this study, we show that among malaria patients an increased IVC collapsibility correlated with clinical signs of hypovolaemia, while a B-pattern on LUS was associated with clinical signs of pulmonary oedema. Therefore, when used at the bedside as an adjunct to the physical examination, IVC US could lead to more rapid and accurate assessment of patients who would most likely benefit from fluid administration, while LUS could help with the detection of early pulmonary oedema and to avoid fluids in these patients. Importantly, other imaging modalities, such as chest X-ray are often not available in malaria settings or detect pulmonary oedema with too much delay as compared to US for preventing clinically relevant disease. In addition, the relative cost-effectiveness, portability of the US device and user-friendliness of POCUS make it an ideal tool for use in resource-limited settings. Future studies should explore whether US measures correlate with additional indicators of volume status, evaluate the relationship between US and fluid responsiveness in malaria patients and investigate the ability of US to improve patient outcomes.

## Supplementary Information

Below is the link to the electronic supplementary material.

Supplementary file1 (PDF 6954 KB)

Supplementary file2 (PDF 53 KB)

## Data Availability

The datasets used and/or analysed during the current study are available from the corresponding author on reasonable request**.**
