# Supplementary material for: Point-of-care ultrasound to assess volume status and pulmonary oedema in malaria patients
Source: Infection. 2021 Jun 10;50(1):65–82. doi: 10.1007/s15010-021-01637-2 (PMC8803774; doi:10.1007/s15010-021-01637-2)
Supplement: Supplementary file 1 — Supplementary file1 (PDF 6954 KB) [file 15010_2021_1637_MOESM1_ESM.pdf]

Article Title: Point-of-care ultrasound to assess volume status and pulmonary oedema in malaria patients

Journal: Infection

Authors: Christina M. Pugliese, Bayode R. Adegbite, Jean R. Edoa, Ghyslain Mombo-Ngoma, Fridia A. Obone-Atome, Charlotte C. Heuvelings, Sabine Bélard, Laura C. Kalkman, Stije J. Leopold, Thomas Hänscheid, Ayola A. Adegnika, Mischa A. Huson, Martin P. Grobusch

Corresponding Author: Prof. M.P. Grobusch, Amsterdam University Medical Centers, email: m.p.grobusch@amsterdamumc.nl

Ultrasound Protocol

Equipment:

- Butterfly IQ™ handheld ultrasound probe
- Compatible mobile device
- Downloaded/installed Butterfly IQ™ App
- Ultrasound gel
- Bed or examination table
- Microfibre cloth/towel or disinfectant wipes

Procedural steps:

Inferior vena cava (IVC) and abdominal aorta

1. Place the subject in a supine position on the bed or exam table. If necessary, small children may be scanned while seated in the caregiver's lap to improve compliance.

2. Connect the probe to the mobile device and open the Butterfly IQ™ App.

3. Tap the 'Pre-sets' icon. Use the 'Cardiac' or 'Pediatric Cardiac' setting for most subjects. In larger adults, the 'Abdominal' pre-set may be used for increased depth.

4. Manually enter the subject ID number by tapping the 'Capture Reel' icon then selecting 'Associate a Patient'.

5. Place a reasonable amount of ultrasound gel onto the probe.

6. Orient the probe in the transverse plane with the probe marker pointing towards the subject's right side. Place the probe in the sub-xyphoid region in the subject's midline.

7. Use the liver as an acoustic window to identify the aorta. It should be visible as a circular structure on the right side of the screen just superior to the vertebral shadow (**Figure 1a**). Once the aorta is in view, tap 'Record' to capture a short video clip (5-10 seconds), then tap 'Stop' to save the video clip.

8. Once the video is saved, tap 'Freeze' to freeze the image. Remove the probe from the subject's abdomen.

9. Tap the 'Review' icon to scroll backwards through the frames. Select the frame that shows the aorta at its maximal width. Tap the 'Measurement Tools' icon then select 'Linear Measurement'. Use the touch screen callipers to measure the anterior-posterior (A-P) aorta diameter from inner wall to inner wall, then tap 'Capture' to save the image (**Figure 1b**).

10. Next, place the probe back on the subject's abdomen just below the subxiphoid process, this time orienting the probe in the longitudinal plane with the probe marker pointing towards the subject's head.

11. Using the liver as an acoustic window, locate the IVC as it leads into the right atrium. If the IVC-hepatic vein junction is not visible, gently fan the probe back and forth until it comes into view.

12. Tap the 'Modes' icon and select 'M-mode'. Use the touch screen marker to adjust the radial scan line so that it intersects the IVC approximately 2 cm distal to the IVC-hepatic vein junction. Freeze the image and remove the probe from the subject's abdomen (**Figure 2a**).

13. Use the 'Linear Measurement' tool to measure the maximal and minimal A-P IVC diameters from inner wall to inner wall. Select 'Capture' to save the image (**Figure 2b**).

14. The IVC collapsibility index will be calculated according to the following formula: ([max IVC–min IVC]/max IVC × 100%). The IVC-to-aorta ratio will be calculated by dividing the maximal A-P diameter of the IVC by the maximal A-P diameter of the aorta.

Figure 1. Transverse view of the abdominal aorta in a paediatric subject.

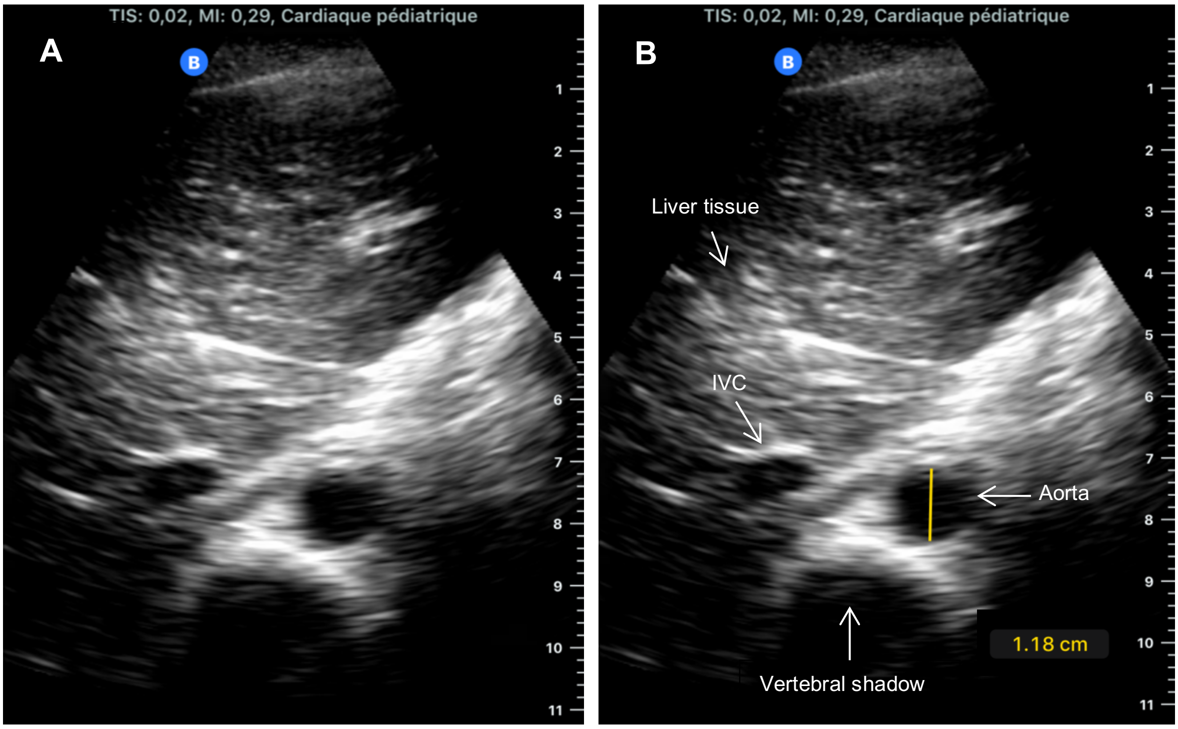

Abbreviations: IVC, inferior vena cava.

Figure 2. M-mode ultrasound measurement of IVC collapse in a paediatric subject.

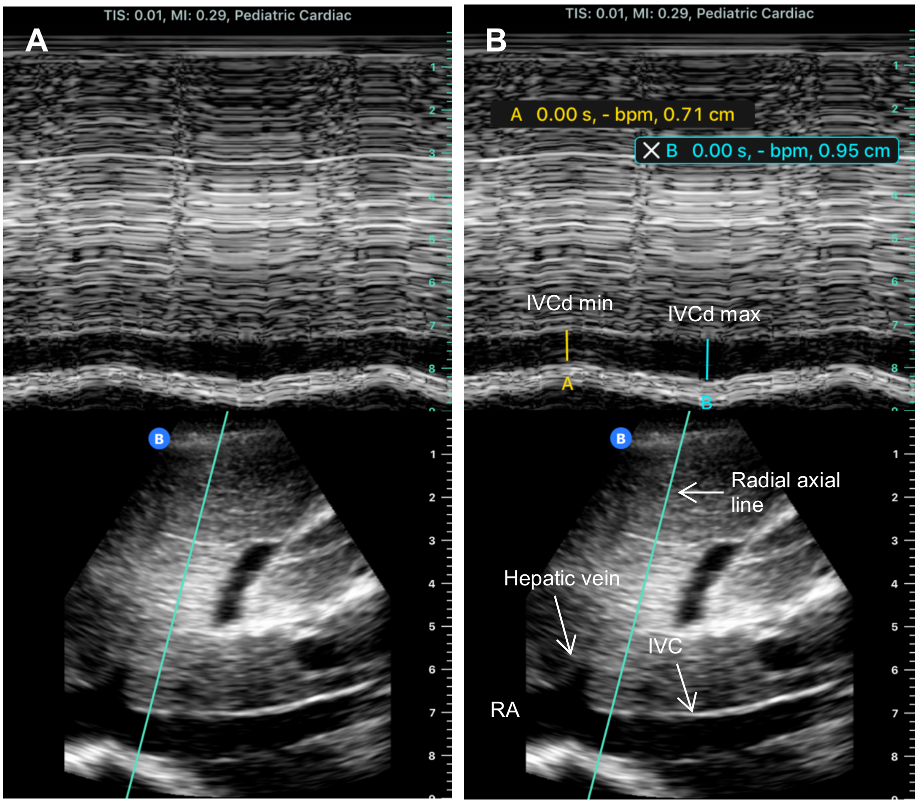

Abbreviations: IVC, inferior vena cava; IVCd min, minimum IVC diameter; IVCd max, maximum IVC diameter; RA, right atrium.

Lung ultrasound (LUS)

1. Tap the ‘Pre-sets’ icon and select ‘Lung’ or ‘Pediatric Lung’, depending on the age of the subject. Place the subject in a supine position on the bed or exam table. If necessary, small children may be scanned while seated in the caregiver’s lap to improve compliance.

Subjects aged 12 years or older

i. Each hemithorax will be divided into 6 scanning zones, (right side: R1 to R6; left side: L1 to L6) covering the upper and lower anterior (e.g. R1, R2), lateral (R3, R4) and dorsolateral (R5, R6) areas of the chest.

ii. Anterior chest: With the subject in a supine position, place a reasonable amount of ultrasound gel onto the probe. Orient the probe in the longitudinal plane with the probe marker pointing towards the subject’s head. Record a short video clip (~10 seconds) in each of the following chest zones: right upper anterior (R1), right lower anterior (R2), left upper anterior (L1), left lower anterior (L2).

iii. Lateral chest: Have the subject lie in a supine position with arms placed above the head. Orient the probe in the longitudinal plane with the probe marker pointing towards the subject’s head. Record a short video clip (~10 seconds) in each of the following chest zones: right upper lateral (R3), right lower lateral (R4), left upper lateral (L3), left lower lateral (L4).

iv. Dorsolateral chest: Next, have the subject switch to a prone position. Orient the probe in the longitudinal plane with the probe marker pointing towards the subject's head. Record a short video clip (~10 seconds) in each of the following chest zones: right upper dorsolateral (R5), right lower dorsolateral (R5), left upper dorsolateral (L5), left lower dorsolateral (L6).

Subject less than 12 years of age

v. The chest will be divided into 12 scanning zones, covering the bilateral upper and lower aspects of the anterior, lateral, and posterior chest.

vi. Right anterior chest: With the subject in a **supine** position, place a reasonable amount of ultrasound gel onto the probe. Orient the probe in the longitudinal plane with the probe marker pointing towards the subject's head. Place the probe in the **mid-clavicular line below the right clavicle**. Record a video clip as the probe is moved caudally from the sub-clavicular region to the diaphragm, scanning every intercostal space systematically. Record a second video clip, repeating the same motion as before but with the probe oriented in the transverse plane. The right anterior chest will be divided into right upper anterior (R1) and right lower anterior (R2) zones to document the findings.

vii. Left anterior chest: Repeat the above step on the left side of the chest. The left anterior chest will be divided into left upper anterior (L1) and left lower anterior (L2) zones to document the findings.

viii. Right lateral chest: Have the subject lie in a **supine** position with the right arm placed above the head. Orient the probe in the longitudinal plane with the probe marker pointing towards the subject's head. Place the probe in the **mid-axillar line in the right axilla**. Record a video clip as the probe is moved caudally down to the diaphragm, scanning every intercostal space systematically. Record a second video clip, repeating the same motion as before but with the probe oriented in the transverse plane. The right lateral chest will be divided into right upper lateral (R3) and right lower lateral (R4) zones to document the findings.

ix. Left lateral chest: Repeat the above step on the left side of the chest. The left lateral chest will be divided into left upper lateral (L3) and left lower lateral (L4) zones to document the findings.

x. Right posterior chest: With the subject in a **prone** position, orient the probe in the longitudinal plane with the probe marker pointing towards the subject's head. Place the probe in the **mid-clavicular line below the right scapula**. Record a video clip as the probe is moved caudally down to the diaphragm, scanning every intercostal space systematically. Record a second video clip, repeating the same motion as before but with the probe oriented in the transverse plane. The right posterior chest will be divided into right upper posterior (R5) and right lower posterior (R6) zones to document the findings.

xi. Left posterior chest: Repeat the above step on the left side of the chest. The left posterior chest will be divided into left upper posterior (L5) and left lower posterior (L6) zones to document the findings.

2. Each lung region will be scored based on four pre-defined aeration patterns of which the most abnormal score will be recorded; sub-pleural nodules and pleural effusions and will be separately noted (**Table 1**; **Figure 3**). Bilateral interstitial syndrome will be defined as having bilaterally ≥2 areas showing a B-pattern (either B1 or B2 pattern).

3. Image quality is documented as 'good' (pleural line, A-lines and/or findings clearly visible in all views), 'moderate' (pleural line, A-lines and/or findings clearly visible in most but not all views), or 'poor' (pleural line, A-lines and/or findings not clearly visible in most views).

4. Participant compliance is documented as 'good' (calm and cooperative), 'moderate' (agitated but remained cooperative) or 'poor' (agitated and uncooperative/moving throughout examination).

5. At the end of the bedside ultrasound examination the gel will be wiped off the patient and the ultrasound probe will be cleaned.

Table 1. Lung aeration patterns

Lung aeration pattern	Description
Normal aeration (A pattern)	Presence of lung sliding with A-lines or fewer than 2 isolated B-lines
Moderate loss of lung aeration (B1 pattern)	Multiple (>2) well-defined B-lines
Severe loss of lung aeration (B2 pattern)	Multiple coalescent B-lines
Lung consolidation (C pattern)	Presence of a sub-pleural, echo-poor, tissue-like area >0.5 cm with or without air bronchograms and causing disruption of the pleural line.
Sub-pleural nodule	Presence of a sub-pleural consolidation <0.5 cm causing disruption of the pleural line
Pleural effusion	Anechoic collection between the pleural line and chest wall

Figure 3. Common LUS findings

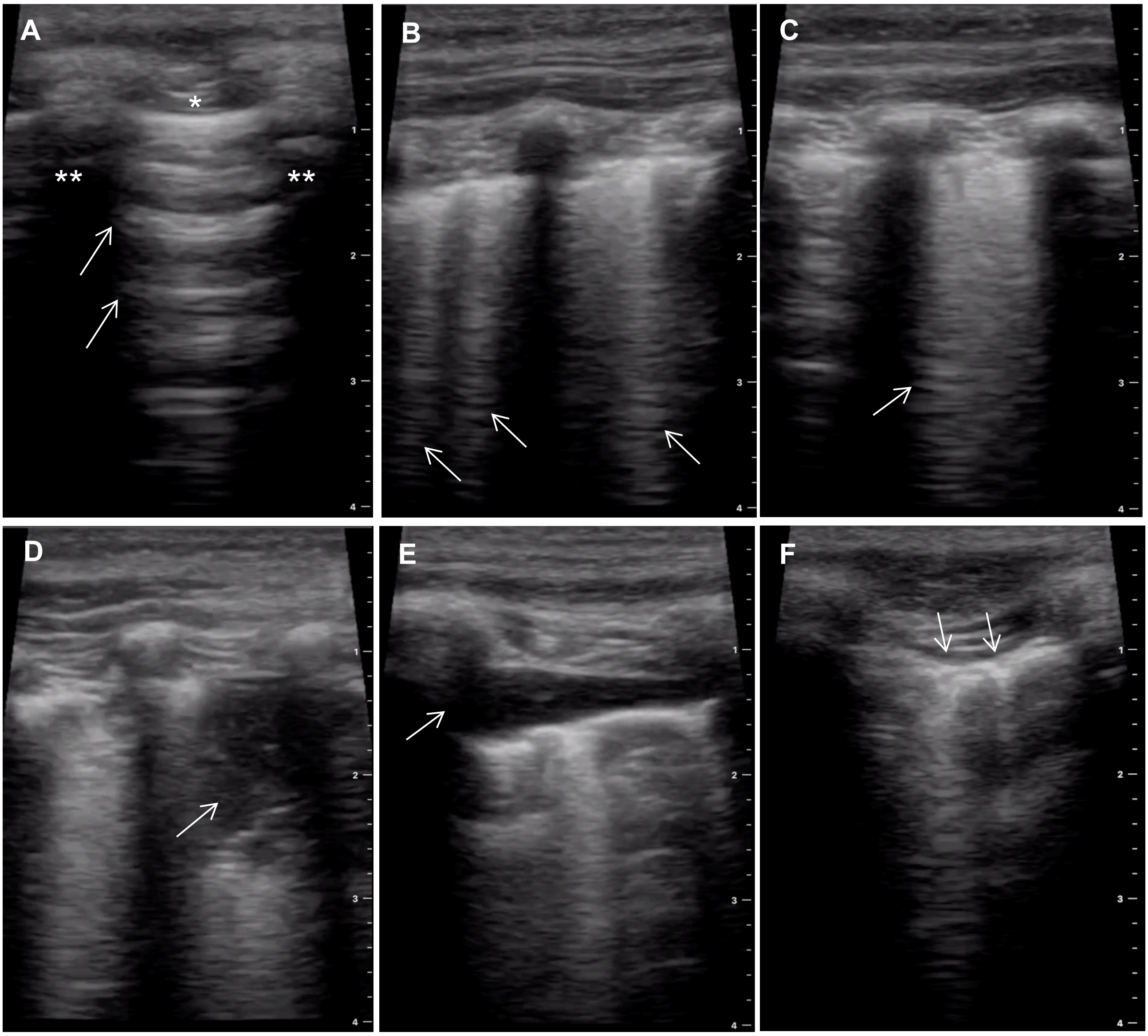

a. A lines (arrows), reverberation artefacts of the pleural line (*) between two rib shadows (**); **b.** Isolated B lines (arrows), pathological signs of interstitial pulmonary oedema; **c.** Multiple coalescing B lines (arrow); **d.** Consolidation (arrow) showing characteristic tissue-like pattern (i.e. “lung hepatization”) and air bronchograms; **e.** Pleural effusion (arrow); **f.** Sub-pleural nodules (arrows).

References

1. Leopold SJ, Ghose A, Plewes K, Mazumder S, Pisani L, Kingston HWF, et al. (2018). Point-of-care ultrasound for the detection of pulmonary manifestations of malaria and sepsis: an observational study. PLoS ONE, 13(12):e0204832.
2. Heuvelings CC, Bélard S, Andronikou S, Jamieson-Luff N, Grobusch MP, Zar HJ. (2019). Chest ultrasound findings in children with suspected pulmonary tuberculosis. Pediatr Pulmonol, 54(4):463-70.
3. Lee FCY. (2016). Lung ultrasound – a primary survey of the acutely dyspneic patient. J Intensive Care, 4:57.
