# Supplementary material for: Point-of-care ultrasound to assess volume status and pulmonary oedema in malaria patients
Source: Infection. 2021 Jun 10;50(1):65–82. doi: 10.1007/s15010-021-01637-2 (PMC8803774; doi:10.1007/s15010-021-01637-2)
Supplement: Supplementary file 2 — Supplementary file2 (PDF 53 KB) [file 15010_2021_1637_MOESM2_ESM.pdf]

Article Title: Point-of-care ultrasound to assess volume status and pulmonary oedema in malaria patients

Journal: Infection

Authors: Christina M. Pugliese, Bayode R. Adegbite, Jean R. Edoa, Ghyslain Mombo-Ngoma, Fridia A. Obone-Atome, Charlotte C. Heuvelings, Sabine Bélard, Laura C. Kalkman, Stije J. Leopold, Thomas Hänscheid, Ayola A. Adegnika, Mischa A. Huson, Martin P. Grobusch

Corresponding Author: Prof. M.P. Grobusch, Amsterdam University Medical Centers, email: m.p.grobusch@amsterdamumc.nl

Supplementary Table 1a. Relationship between IVC US measures and clinical signs of hypovolaemia on days 1-2-3 in malaria patients.

Clinical finding		N	IVC-CI				IVC/Ao			
			≥50%, n (%)	*p*	rho	*p*	≤0.8, n (%)	*p*	rho	*p*
Eyes	Sunken	9	2 (22)	.53	–0.00	.49	3 (33)	.58	–0.02	.43
	Normal	77	14 (18)				24 (31)			
CRT	Prolonged	12	1 (8)	.30	–0.01	.46	4 (33)	.56	0.03	.38
	Normal	74	15 (20)				23 (31)			
HR	Tachycardia [1]	5	3 (60)	.044	0.26	.0086*	5 (100)	.003	–0.30	.0028*
	No tachycardia	80	13 (16)				22 (28)			
Respiratory effort	Increased	23	6 (26)	.22	0.25	.011*	14 (61)	.0006	–0.37	.0003*
	Normal	63	10 (16)				13 (21)			
RR	Tachypnoea	37	5 (14)	.22	0.11	.17	13 (35)	0.34	–0.08	.22
	Normal	49	11 (22)				14 (29)			

Chi-square test or Fisher's exact test for categorical variables. Spearman's rank for correlation coefficients. *Indicates Spearman correlation that remained statistically significant after the Holm-Sidak correction. Abbreviations: CRT, capillary refill time; HR, heart rate; IVC-CI, Inferior vena cava collapsibility index; IVC/Ao, Inferior vena cava-to-aorta ratio; rho, Spearman's correlation coefficient; RR, respiratory rate. [1]As defined in the Methods section.

Supplementary Table 1b. Relationship between abnormal LUS patterns and clinical signs of pulmonary oedema on days 1-2-3 in malaria patients.

Clinical finding		N	B pattern				C pattern			
			≥1 area, n (%)	*p*	rho	*p*	≥1 area, n (%)	*p*	rho	*p*
Respiratory effort	Increased	24	3 (13)	0.058	0.23	.013	11 (46)	<.0001	0.50	<.0001*
	Normal	65	1 (2)				3 (5)			
Lung auscultation	Crepitations	13	1 (8)	0.47	0.06	.28	7 (54)	.0005	0.43	<.0001*
	Normal	76	3 (4)				7 (9)			
RR	Tachypnoea[1]	39	1 (3)	0.41	–0.08	.22	9 (23)	.083	0.18	.047
	Normal	50	3 (6)				5 (10)			
SpO2	≤ 94%	5	0 (0)	0.78	–0.05	.31	1 (20)	.58	0.02	.42
	> 94%	79	4 (5)				13 (15)			

Chi-square test or Fisher's exact test for categorical variables. Spearman's rank for correlation coefficients. *Indicates Spearman correlation that remained statistically significant after the Holm-Sidak correction. Abbreviations: rho, Spearman's correlation coefficient; RR, respiratory rate; SpO2, oxygen saturation. [1]As defined in Methods section.
